# Palladium–Platinum Alloy Nanoribbon/Nanosheets for Room-Temperature Hydrogen Detection

**DOI:** 10.3390/s26196231

**Published:** 2026-09-30

**Authors:** Sadaf Mohsenifard, Thomas Thundat, Mark T. Swihart

**Affiliations:** 1 Department of Chemical and Biological Engineering, University at Buffalo (SUNY), Buffalo, NY 14260, USA; sadafmoh@buffalo.edu (S.M.); tgthunda@buffalo.edu (T.T.); 2 RENEW Institute, University at Buffalo (SUNY), Buffalo, NY 14260, USA

**Keywords:** palladium nanosheet, nanoribbon, hydrogen gas sensor, platinum, sensing materials

## Abstract

Hydrogen is being extensively investigated as a carbon-free energy carrier, particularly for fuel-cell vehicles and long-term energy storage. Because H_2_ can form explosive mixtures in air at concentrations as low as 4%, its safe use requires inexpensive, low-power sensors capable of reliable detection. Here, we investigated ribbon-like palladium–platinum nanostructures, referred to as PdPt nanoribbon/nanosheet hybrid structures (PdPt NR/NSs), for room-temperature chemiresistive H_2_ sensing. Sensors were fabricated by drop-casting dispersions of the PdPt NR/NSs onto interdigitated electrodes. The sensor resistance increased upon exposure to H_2_ because of hydrogen uptake by the PdPt NR/NSs and formation of a hydride phase with higher resistivity than the hydrogen-free alloy. The best-performing sensor, Pd_5_Pt_8_, was prepared using a 5:8 Pd:Pt precursor mass ratio, which yielded a measured Pd:Pt atomic ratio of approximately 1.3:1. This sensor showed a response time of 16 s and a response of 6.0% to 1 vol% H_2_ in air. H_2_ concentrations as low as 100 ppm were readily detected while maintaining a positive resistance response. These results demonstrate the potential of PdPt NR/NS architectures for low-power H_2_ detection at ambient temperature.

## 1. Introduction

Hydrogen (H_2_) is a clean energy carrier with significant potential for transportation and energy-storage applications because of its high gravimetric energy density and the fact that its combustion or reaction in a fuel cell produces only water [1]. However, hydrogen is a colorless and odorless gas with a lower explosive limit of approximately 4 vol% in air [2]. Therefore, reliable detection of hydrogen leaks is essential for its safe use, particularly as hydrogen technologies expand beyond controlled industrial environments. The U.S. Department of Energy (DOE) has established performance targets for hydrogen sensors, including rapid response times of less than 1 s over a broad concentration range of approximately 0.1–10 vol% H_2_ [3].

Chemiresistive gas sensors detect target gases by measuring changes in the electrical resistance of a sensing material. For H_2_ sensing, hydrogen adsorbs on the sensing-material surface and may subsequently diffuse into the material, altering its intrinsic conductivity and/or the contact resistance within the sensing network [2,4,5,6]. Compared with optical or more complex electronic sensing approaches, chemiresistive sensors offer simple electrical readout, relatively low power consumption, and straightforward integration into compact devices and sensor arrays [7,8,9]. These advantages, together with their potential sensitivity to low H_2_ concentrations, make chemiresistive sensors attractive for practical hydrogen-detection applications and form the basis for the sensing approach investigated in this work.

Transition-metal oxides are widely investigated for chemiresistive H_2_ sensing because of their low cost, chemical stability, and compatibility with scalable fabrication [4]. However, most metal-oxide-based sensors require elevated operating temperatures to achieve sufficiently fast surface-reaction kinetics, which increases power consumption and can complicate portable or continuously operated sensing applications [1,5]. In contrast, noble metals such as Pd and Pt can adsorb and dissociate H_2_ to enable sensitive H_2_ detection at or near room temperature [1]. Pd is particularly attractive because absorbed hydrogen forms palladium hydride (PdH_x_), producing a measurable change in electrical resistance, while Pt can promote H_2_ dissociation and accelerate sensing kinetics. These complementary properties make Pd–Pt materials attractive candidates for low-power, room-temperature H_2_ sensing [1].

Palladium (Pd) has been extensively studied for H_2_-sensing applications because of its ability to absorb hydrogen and form palladium hydride at room temperature, producing a measurable increase in electrical resistance [10]. Pd-based sensors can also exhibit useful selectivity toward H_2_ relative to common interfering gases, although the degree of cross-sensitivity depends on sensor architecture and operating conditions [11]. Consequently, a wide variety of Pd-based H_2_ sensors have been developed, and this remains an active area of research [1,12,13]. 

In addition to sensing-material composition, the dimensionality and morphology of Pd-based structures strongly influence H_2_-sensing performance. Pd-based H_2_ sensors have been investigated in several architectures, including conventional thin films, one-dimensional nanowires (NWs), and two-dimensional nanosheets (NSs), each of which offers distinct advantages. Pd-based thin films are widely studied because of their strong interaction with hydrogen and compatibility with conventional microfabrication processes. Nanowires are attractive for chemiresistive sensing because their high surface-to-volume ratio provides abundant sites for gas interaction, while their small dimensions provide short diffusion pathways for hydrogen. Their one-dimensional geometry also provides direct electrical conduction pathways and facilitates integration into flexible, paper-based, and wearable devices [14]. We have previously shown that ultrathin Pd NWs can provide high H_2_ sensitivity, with thinner nanowires (<5 nm) exhibiting faster and stronger responses because of shorter hydrogen-diffusion distances and a greater fraction of surface and low-coordination sites [15]. Pd nanowires also provide a tunable sensing platform: they can be synthesized in polycrystalline or single-crystalline forms [10], modified with Pt to accelerate hydrogen dissociation [16], or combined with supporting materials such as paper, reduced graphene oxide, or carbon nanotubes to form hybrid sensors.

Two-dimensional (2D) nanosheet (NS) structures are attractive for gas-sensing applications because their high surface-to-volume ratio provides abundant sites for H_2_ interaction, while their small thickness provides short diffusion pathways for absorbed hydrogen [11,17,18,19,20,21,22,23]. Their extended lateral dimensions can also provide relatively large inter-sheet contact areas, which may improve electrical connectivity within a sensing network. These features make nanosheet architectures promising for room-temperature H_2_ sensing [7,12,24,25,26,27,28]. In a previous study from our group, Pd nanosheets (PdNSs) were used to develop low-cost, room-temperature sensors for H_2_ and carbon monoxide (CO) detection [11]. Pure PdNS sensors showed limited H_2_ response, whereas incorporation of multiwalled carbon nanotubes (MWCNTs) significantly improved sensing performance. The MWCNT@PdNS composite exhibited measurable H_2_ detection down to 5 ppm and showed increased sensitivity with increasing MWCNT content. The sensors also demonstrated repeatability and stability under humid conditions and exhibited preferential response toward H_2_ relative to CH_4_, CO_2_, and NH_3_. In contrast, CO produced a response of opposite sign, which dominated when H_2_ and CO were present simultaneously [11]. These results demonstrated the potential of PdNS-based nanostructured networks for room-temperature gas sensing.

Pd can be combined with other metals to modify its electronic structure and catalytic activity [29,30,31]. In particular, alloying or combining Pd with Pt can enhance hydrogen dissociation kinetics. In our previous work, decorating penta-twinned Pd nanowires with a small amount of Pt (<5%) reduced the H_2_ response and recovery times to approximately 5 s by promoting hydrogen dissociation [16]. However, devices prepared by simply drop-casting these nanowires onto interdigitated electrodes or paper substrates exhibited a negative resistance response at low H_2_ concentrations. This behavior was attributed to changes in contact resistance between ligand-coated nanowires: hydride-induced volume expansion increased the contact area or reduced the gaps between neighboring nanowires, thereby decreasing the overall device resistance. At higher H_2_ concentrations, the increasing intrinsic resistivity of the Pd hydride could dominate, producing non-monotonic sensing behavior. In that study, UV-ozone treatment was used to degrade the surface PVP and eliminate the negative response [16]. Although effective, avoiding such an additional post-treatment step would simplify sensor fabrication. These previous results therefore highlight the strong influence of both composition and nanostructure morphology on H_2_-sensing behavior and motivate the development of solution-processed Pd-based architectures that provide rapid, monotonic positive resistance responses without post-synthesis treatment.

Pd nanosheets have been synthesized using several approaches, including chemical vapor deposition, polyol and solvothermal methods, chemical reduction, electrodeposition, galvanic replacement, and template-assisted synthesis [11,32,33,34,35,36]. More recently, Sun et al. reported a wet-chemical coreduction method for the synthesis of ultrathin PdPtH nanosheets [37]. In this approach, Pd and Pt precursors are simultaneously reduced in oleylamine using CO generated from Mo(CO)_6_ decomposition. The CO also acts as a capping agent, preferentially stabilizing the top and bottom surfaces of the growing 2-dimensional structures and promoting the formation of ultrathin nanosheets or nanoribbons (NSs/NRs) rather than nanoparticles [37].

The NS/NR morphology provides a high surface-to-volume ratio, short cross-sheet hydrogen-diffusion pathways, extended in-plane electrical transport pathways, and relatively large inter-sheet contact areas compared with nanowire networks. In addition, Pt incorporation can promote H_2_ dissociation and thereby influence sensing kinetics. To the best of our knowledge, this is the first use of this type of PdPt alloy NR/NS architecture for H_2_ sensing. Previous studies of related PdPt materials have focused primarily on catalytic applications [37], while our earlier H_2_-sensing studies employed Pt-decorated Pd nanowires rather than alloyed PdPt NR/NS structures [16]. In the present study, we systematically examine the influence of Pd/Pt composition on sensor performance and characterize the materials with respect to morphology, crystal structure, and elemental composition. We further evaluate H_2_ response over a range of concentrations, as well as the effects of sensor loading, humidity, interfering gases, response and recovery behavior, stability, and reproducibility. Finally, the sensing mechanism is discussed in relation to hydrogen adsorption, dissociation, absorption, hydride formation, and Pt incorporation.

## 2. Materials and Methods

**Figure 1:**
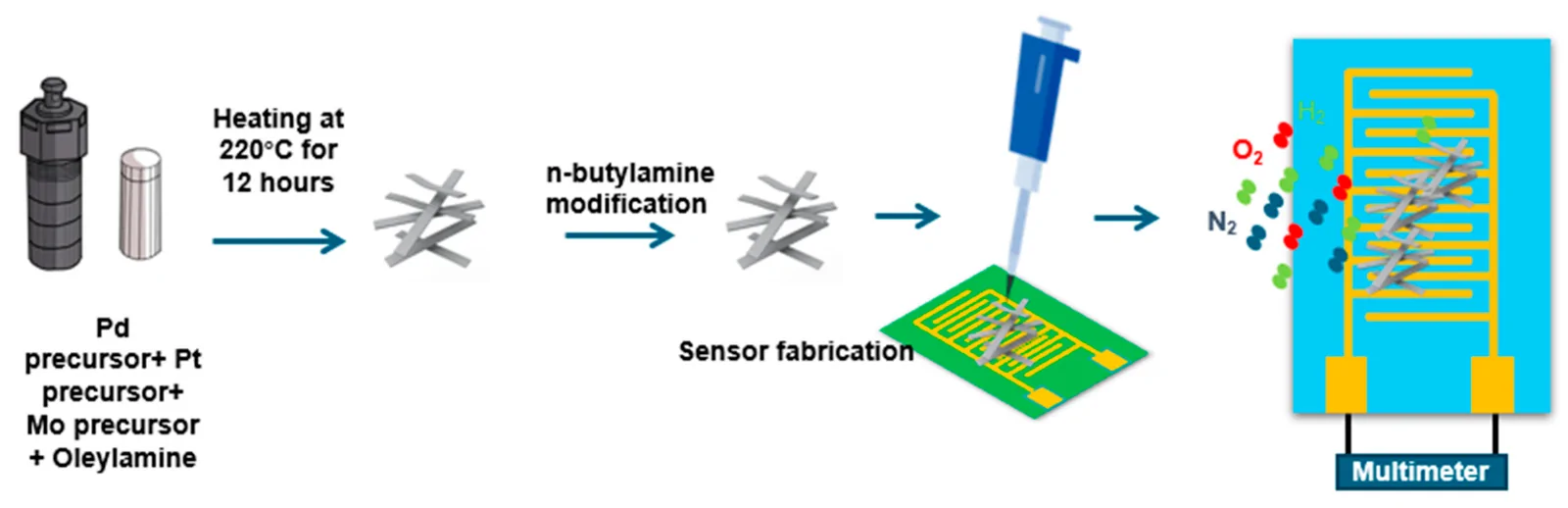
Schematic illustration of the preparation and testing of PdPt NR/NS IDE-based sensors.

The overall process of PdPt NR/NS synthesis, sensor fabrication, and testing is illustrated schematically in Figure 1.

### 2.1. Materials

Platinum(II) acetylacetonate (Pt(acac)_2_, 98%, Thermo Scientific, Waltham, MA, USA), palladium(II) acetylacetonate (Pd(acac)_2_, 99%, Sigma-Aldrich, St. Louis, MO, USA/Umicore, Brussels, Belgium), oleylamine (technical grade, 70%, Thermo Scientific, Waltham, MA, USA), *n*-butylamine (99+%, Thermo Scientific, Waltham, MA, USA), and molybdenum hexacarbonyl (Mo(CO)_6_, 98%, Thermo Scientific, Waltham, MA, USA) were used as received. Ethanol (200 proof, anhydrous, Decon Laboratories, King of Prussia, PA, USA) and hexanes (BAKER ANALYZED^®^ A.C.S. Reagent, J.T. Baker/Avantor, Radnor, PA, USA) were used as solvents.

### 2.2. Synthesis of Pd and PdPt NR/NSs

The synthesis followed the general procedure reported by Sun et al. [37]. In a typical synthesis of PdPt NR/NSs using an 11:2 Pd:Pt precursor mass ratio, 2 mg (0.005 mmol) of Pt(acac)_2_, 11 mg (0.036 mmol) of Pd(acac)_2_, and 35 mg (0.13 mmol) of Mo(CO)_6_ were sequentially added to a 25 mL Teflon-lined stainless-steel autoclave containing 10 mL of oleylamine. A clear orange solution was obtained after 10 min of bath sonication. The autoclave was heated from 40 °C to 220 °C over approximately 60 min and maintained at 220 °C for 12 h. It was then allowed to cool to room temperature. The products were collected by centrifugation at 10,000 rpm for 5 min and washed several times with a 1:3 (*v*/*v*) mixture of *n*-hexane and ethanol to remove impurities. The sample was then dispersed in *n*-butylamine and stirred for 24 h to promote ligand exchange, allowing *n*-butylamine to displace oleylamine from the NR/NS surface. Finally, the sample was dispersed in ethanol at a concentration of 10 mg/mL for subsequent characterization and sensor fabrication.

The same synthesis procedure was used for the other compositions by adjusting the masses of Pd(acac)_2_ and Pt(acac)_2_. Precursor Pd:Pt mass ratios of 11:2, 9:4, 7:6, and 5:8 correspond to approximate Pd:Pt molar ratios of 7:1, 3:1, 1.5:1, and 0.8:1, respectively. The resulting samples, and the sensors fabricated from them, were designated Pd_11_Pt_2_, Pd_9_Pt_4_, Pd_7_Pt_6_, and Pd_5_Pt_8_ according to the precursor quantities used. Samples with Pd:Pt precursor mass ratios of 13:0 (pure Pd) and 3:10 were also synthesized and characterized; however, the Pd_3_Pt_10_ composition did not provide a useful response during initial H_2_-sensing tests and was therefore not investigated further.

### 2.3. Sensor Fabrication 

PdPt NR/NS sensors were fabricated by a simple drop-casting method. For each sensor, 10 µL of a 10 mg/mL PdPt NR/NS dispersion in ethanol was drop-cast onto an interdigitated electrode (IDE; Micrux Technologies, Gijón, Spain, part ED-IDE1-Au) containing 90 pairs of circular gold fingers with 10 µm linewidth and 10 µm spacing. The deposited material was then dried in a fume hood before sensing measurements. 

### 2.4. Materials Characterization 

The morphologies of the Pd and PdPt NR/NS samples were examined using a JEOL JEM-2010 transmission electron microscope (TEM) operated at an accelerating voltage of 200 kV. For TEM and high-resolution TEM (HRTEM) analysis, the samples were diluted in ethanol, drop-cast onto 200-mesh copper TEM grids with a carbon support film, and dried in a fume hood.

Scanning electron microscopy (SEM) images of the Pd and PdPt NR/NS samples and the corresponding IDE-based sensors were obtained using a Zeiss Crossbeam focused ion beam-scanning electron microscope operated at an accelerating voltage of 2 kV and a working distance of 5 mm. Energy-dispersive X-ray spectroscopy (EDS) was performed using an Oxford Instruments EDX detector attached to the SEM.

The crystal structures of the Pd and PdPt NR/NS samples were characterized using a Rigaku Ultima IV X-ray diffractometer with Cu Kα radiation (λ = 1.54 Å) at a scan rate of 4°/min.

### 2.5. H_2_ Sensing Measurements

For H_2_ sensing measurements, the prepared sensors were mounted onto a NEXTRON probe station, and two of the four gold probes were used to record changes in electrical resistance using a Keithley digital multimeter (Keithley Instruments, Solon, OH, USA, model DMM 6500). For resistance measurements, a constant current of 10 μA to 100 μA was applied, depending upon the sample resistance, to maintain accurate voltage readings with minimal self-heating. For example, a device with 15 Ω resistance and a 100 μA current yields power dissipation of 0.15 μW, while a device with 1 kΩ resistance and a 10 μA current yields power dissipation of 0.10 μW. For dynamic H_2_ sensing measurements, a total flow of H_2_ diluted in air (1 to 0.01 vol %) through the chamber was maintained at 1000 sccm for all measurements, using mass flow controllers (MKS). For concentrations from 1000 ppm up to 10,000 ppm (1%) H_2_ in air, desired concentrations were obtained by dynamic mixing of a stream of 2% H_2_ in air (Airgas) with a stream of dry air (breathing grade, Airgas B300). Similarly, for H_2_ concentrations of 1000 ppm or less, the desired concentrations were obtained by mixing a stream of 1000 ppm H_2_ in air (Airgas) with a stream of dry air. Unless otherwise noted, all H_2_ sensing tests were conducted at 25 °C under dry conditions (nominally 0% relative humidity). Prior to sensing measurements, the chamber was purged with dry air until the sensor resistance reached a stable baseline. For H_2_ sensing measurements, varying concentrations of H_2_ diluted in air (0.01 to 1 vol % or 100 ppm to 10,000 ppm) were employed, each followed by purging with 1000 sccm dry air. The total flow through the chamber was maintained at 1000 sccm throughout, and the interval between H_2_ exposures was 1 min. The sensing chamber had a volume of approximately 100 cm^3^. At a total gas flow rate of 1000 sccm, the nominal gas residence time, defined as the chamber volume divided by the volumetric flow rate, was approximately 6 s. Thus, one chamber volume was replaced approximately every 6 s, which is shorter than the measured response and recovery times in this study. The response of the sensor was evaluated as [11]:

(1) $$\mathrm{Response}\left(\%\right)=\frac{{(R}_{g}-R_{0})}{R_{0}}\times 100$$

where *R*_0_ and *R_g_* are the resistances of the sensors before and after exposure to H_2_ gas. 

## 3. Results and Discussion

### 3.1. Nanoribbon/Nanosheet Synthesis and Characterization

Our synthesis approach followed a wet-chemical coreduction method previously reported to produce PdPt nanostructures containing interstitial hydrogen [37]. For the H_2_-sensing application investigated here, the initial hydrogen content of the as-synthesized PdPt material is not critical because the sensing process involves repeated hydrogen uptake and release during exposure and recovery cycles. The synthesized two-dimensional structures typically exhibited a ribbon-like morphology, with one lateral dimension longer than the other and both lateral dimensions substantially greater than the thickness. Accordingly, these materials are referred to throughout this work as PdPt nanoribbon/nanosheets (NR/NSs).

During synthesis, Mo(CO)_6_ and Pd and Pt acetylacetonate precursors were reacted under solvothermal conditions in oleylamine. Thermal decomposition of Mo(CO)_6_ generates CO, which serves as both a reducing and capping agent during NR/NS formation [37]. Oleylamine serves as the solvent and contributes to reduction and colloidal stabilization of the growing nanostructures. The Teflon-lined autoclave provides a sealed high-temperature environment that retains the CO generated during Mo(CO)_6_ decomposition [37]. Figure 1 summarizes the synthesis of the Pd and PdPt NR/NS materials, their deposition onto interdigitated electrodes by drop-casting, and subsequent H_2_-sensing measurements.

**Figure 2:**
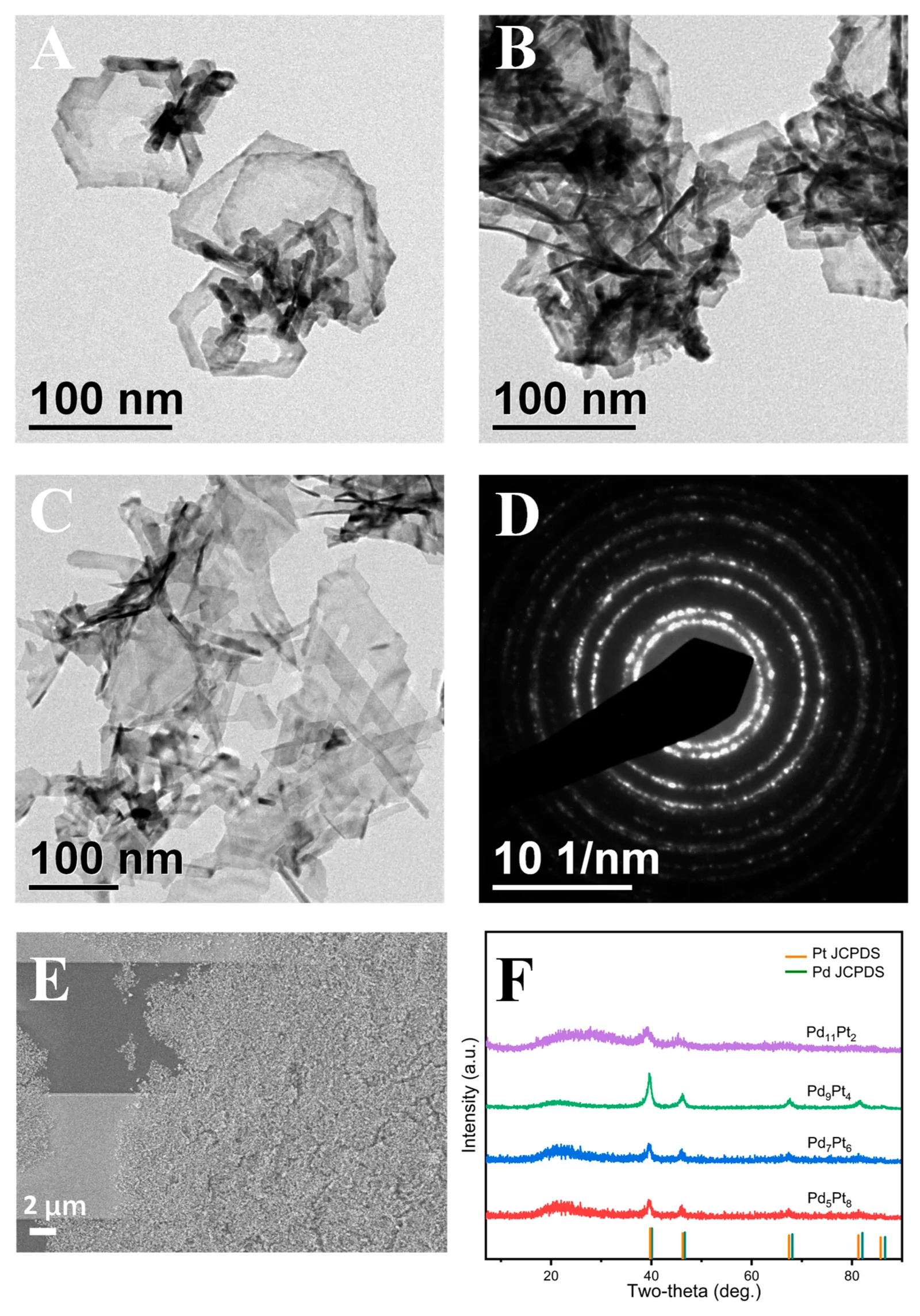
Representative TEM images of (**A**) Pd_5_Pt_8_, (**B**) Pd_11_Pt_2_, and (**C**) Pd_13_Pt_0_ NR/NSs. (**D**) SAED pattern of Pd_13_Pt_0_ NR/NSs. (**E**) Representative SEM image of Pd_11_Pt_2_ NR/NSs on an IDE. (**F**) XRD patterns of Pd NR/NSs and PdPt NR/NSs as indicated.

The morphologies of the Pd and PdPt NR/NS samples were examined by TEM. Samples containing different amounts of Pt exhibited similar two-dimensional ribbon-like morphologies, as shown in Figure 2A–C for Pd_5_Pt_8_, Pd_11_Pt_2_, and Pd_13_Pt_0_, respectively. Figure 2D shows the selected-area electron diffraction (SAED) pattern of the Pd_13_Pt_0_ sample. The pattern consists of spotty diffraction rings characteristic of a polycrystalline material containing relatively large, well-ordered crystallites. The observed rings can be indexed to the (111), (200), (220), and (311) planes of the face-centered cubic (fcc) Pd structure. Figure 2E shows a representative SEM image of Pd_11_Pt_2_ NR/NSs deposited across an interdigitated electrode, demonstrating that the nanostructures bridge adjacent electrode fingers and form an electrically connected sensing network.

The crystal structures of the Pd and PdPt NR/NS samples were further examined by XRD. Figure 2F compares the XRD patterns of Pd_11_Pt_2_ and Pd_13_Pt_0_ with the reference pattern for fcc Pd (JCPDS card No. 00-046-1043). The diffraction peaks observed at 40.12°, 46.66°, 68.12°, 82.10°, and 86.62° can be indexed to the (111), (200), (220), (311), and (222) planes, respectively. No substantial shift in peak position was observed for the PdPt NR/NS sample relative to Pd, which is consistent with the very similar lattice parameters of fcc Pd and fcc Pt. A weak amorphous background was also observed and is attributed to residual organic species on the NR/NS surfaces.

**Figure 3:**
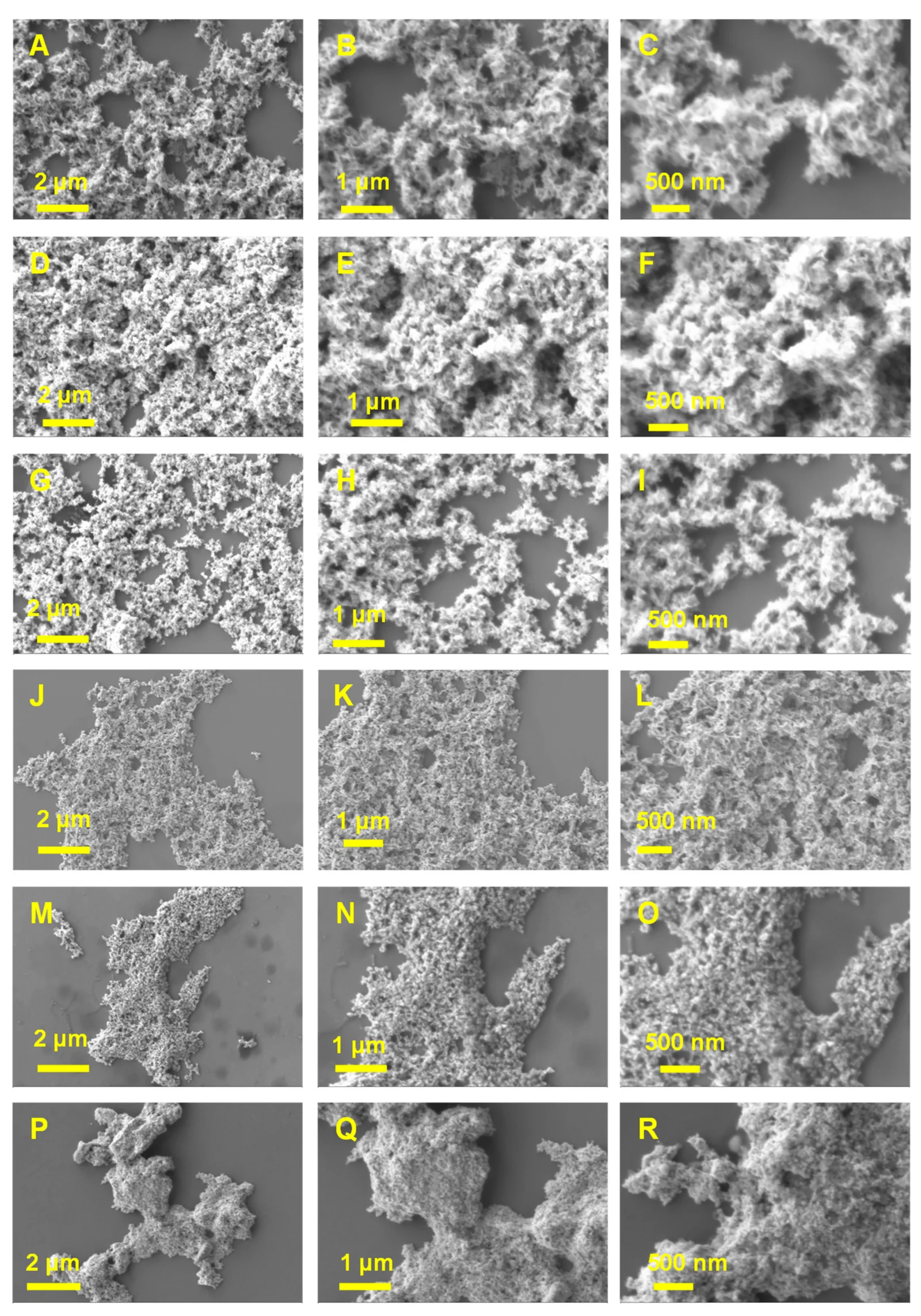
SEM images of samples Pd_13_Pt_0_ (**A**–**C**), Pd_11_Pt_2_ (**D**–**F**), Pd_9_Pt_4_ (**G**–**I**), Pd_7_Pt_6_ (**J**–**L**), Pd_5_Pt_8_ (**M**–**O**), and Pd_3_Pt_10_ (**P**–**R**).

Representative SEM images of all compositions at three different magnifications are shown in Figure 3. The pure Pd sample, Pd_13_Pt_0_, exhibits an aggregated flake-like NR/NS morphology (Figure 3A–C). A similar morphology is retained upon Pt incorporation in Pd_11_Pt_2_ (Figure 3D–F), Pd_9_Pt_4_ (Figure 3G–I), Pd_7_Pt_6_ (Figure 3J–L), and Pd_5_Pt_8_ (Figure 3M–O) [37,38,39]. At the highest Pt loading investigated, however, the aggregated flake-like morphology is no longer clearly evident for Pd_3_Pt_10_ (Figure 3P–R). Because Pd_3_Pt_10_ did not exhibit a useful response during initial H_2_-sensing measurements, it was not characterized further, and subsequent analysis focused on the compositions that provided useful sensing performance. 

EDS elemental maps and quantitative composition analyses for the Pd and PdPt NR/NS samples are provided in Figures S1–S6 of the Supporting Information. The elemental maps showed generally homogeneous distributions of Pd and Pt within the nanostructures, while the quantitative EDS results showed an overall increase in the Pt/Pd ratio with increasing Pt precursor loading, although some sample-to-sample variation was observed. For example, the measured Pd:Pt atomic ratios were approximately 1.3:1 for Pd_5_Pt_8_ and 11.5:1 for Pd_11_Pt_2_.

TGA/DSC analysis of a representative Pd_5_Pt_8_ NR/NS sample is provided in Figure S7 of the Supporting Information. The sample exhibited an overall mass loss of approximately 14% under N_2_, which is attributed primarily to adsorbed species and residual surface-bound organic compounds remaining from the synthesis.

### 3.2. Sensor Response Testing

**Figure 4:**
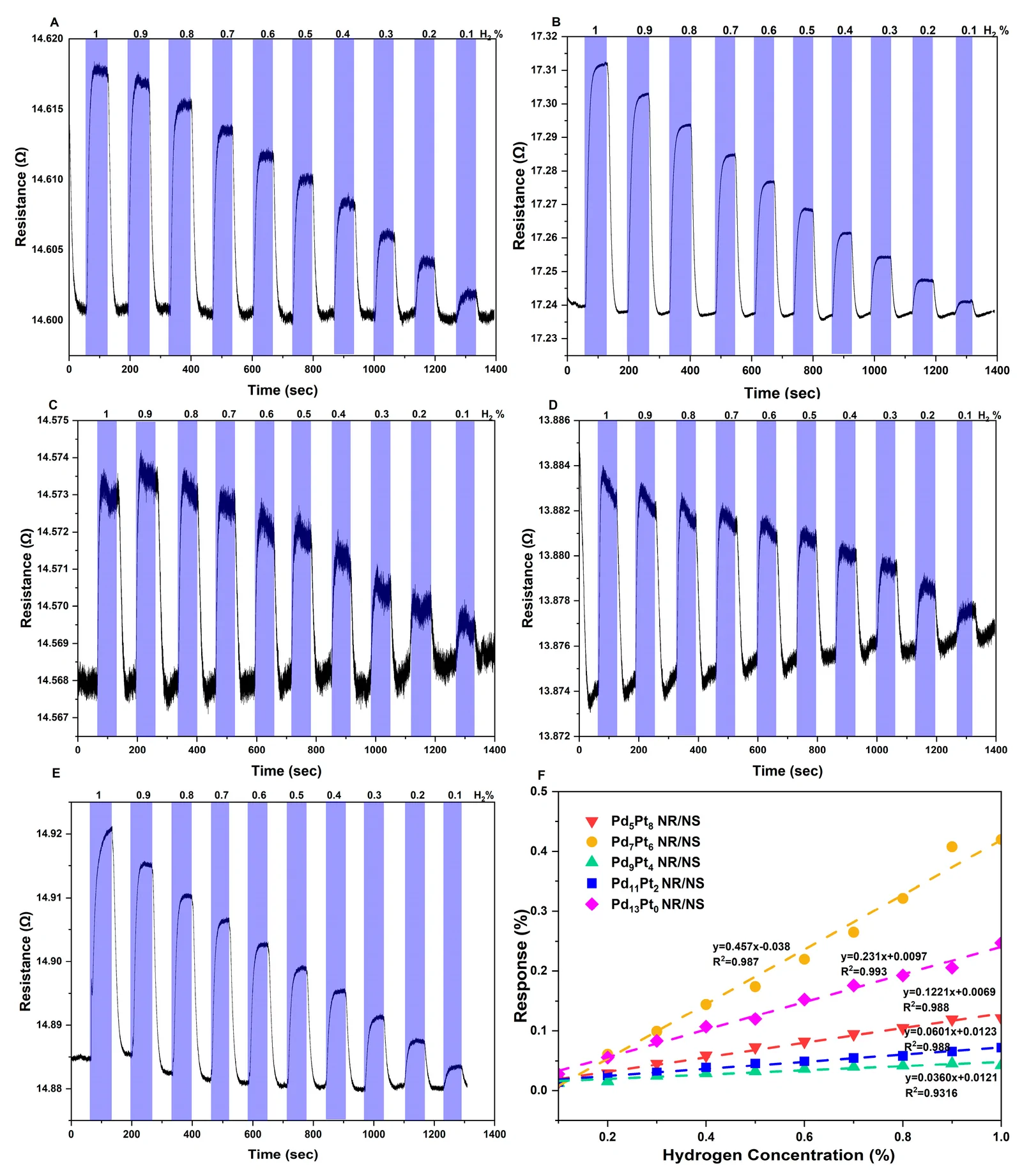
Dynamic H_2_ sensor performance of PdPt NR/NS IDE-based sensors to 1.0–0.1 vol% H_2_ in air at 25 °C for samples (**A**) Pd_5_Pt_8_, (**B**) Pd_7_Pt_6_, (**C**) Pd_9_Pt_4_, (**D**) Pd_11_Pt_2_, and (**E**) Pd_13_Pt_0_. Purple shaded regions indicate approximately the time periods when H_2_ was flowing at the indicated concentration. (**F**) Comparison of the response as a function of H_2_ concentration for these samples. Dashed lines in panel F represent linear fits to the experimental data.

Figure 4 shows the dynamic resistance response of the PdPt NR/NS IDE-based sensors to H_2_ concentrations ranging from 1.0 to 0.1 vol% in air at 25 °C. All investigated compositions exhibited an increase in resistance upon exposure to H_2_. When H_2_ reaches the Pd-containing surface, it dissociates into atomic hydrogen, which can diffuse into the fcc Pd-rich lattice and form α-PdH_x_ (x < 0.017) or β-PdH_x_ (x > 0.58) phases [40]. Hydride formation increases the electrical resistivity of the Pd-containing material and is accompanied by lattice expansion [40]. In networks of discrete Pd nanostructures, however, this volume expansion can also increase the contact area or reduce gaps between neighboring structures, thereby decreasing the overall device resistance and producing a negative response [11]. Such contact effects can result in non-monotonic sensing behavior if they compete with the increase in intrinsic resistivity associated with hydride formation.

In the present PdPt NR/NS sensors, the resistance increased upon H_2_ exposure at all concentrations tested, indicating that the response was dominated by the intrinsic resistance change in the sensing material rather than by a decrease in interparticle contact resistance. This positive response was obtained despite the presence of residual surface-bound organic species indicated by TGA and without post-synthesis treatment of the sensors. This behavior contrasts with our previously reported Pd nanowire sensors, for which UV-ozone treatment was required to remove or degrade surface ligands and obtain a consistently positive resistance response.

However, a positive resistance response is not unique to the present NR/NS sensors, as conventional continuous Pd- and PdPt-based thin-film sensors also generally exhibit positive responses to H_2_. In continuous films, the electrical response is primarily governed by the increase in intrinsic resistivity associated with hydrogen uptake and hydride formation, without the same contribution from changing interparticle contacts that can occur in networks of discrete nanostructures [26,28,41]. Therefore, the advantage of the present NR/NS architecture is not the positive response alone, but rather the ability to retain a monotonic positive response in a simple drop-cast nanostructured network without requiring post-treatment.

Figure 4A–E show the dynamic H_2_ responses of representative Pd_5_Pt_8_, Pd_7_Pt_6_, Pd_9_Pt_4_, Pd_11_Pt_2_, and Pd_13_Pt_0_ sensors, respectively. For the devices shown in Figure 4, the response times, defined as the time required to reach 90% of the full response, were 16, 17, 25, 24, and 32 s, respectively. Some variation in absolute response time and response magnitude was observed among independently fabricated electrodes, although the overall composition-dependent trends were reproducible.

Figure 4F compares the sensor response as a function of H_2_ concentration for these five representative devices. Pd_5_Pt_8_ and Pd_7_Pt_6_ exhibited relatively large responses together with rapid response times. Pure Pd (Pd_13_Pt_0_) also exhibited a substantial response but responded more slowly than the Pt-containing compositions, consistent with the role of Pt in promoting H_2_ dissociation. Linear regression of the response as a function of H_2_ concentration over the 0.1–1.0 vol% range showed strong concentration dependence, with R^2^ values of 0.988, 0.987, 0.932, 0.988, and 0.993 for Pd_5_Pt_8_, Pd_7_Pt_6_, Pd_9_Pt_4_, Pd_11_Pt_2_, and Pd_13_Pt_0_, respectively. Based on the combined response magnitude and response time, Pd_5_Pt_8_ was selected as the representative optimized composition for subsequent evaluation. Pd_3_Pt_10_ was also fabricated into sensors; however, these devices exhibited high resistance with no measurable change upon H_2_ exposure and were therefore not investigated further.

**Figure 5:**
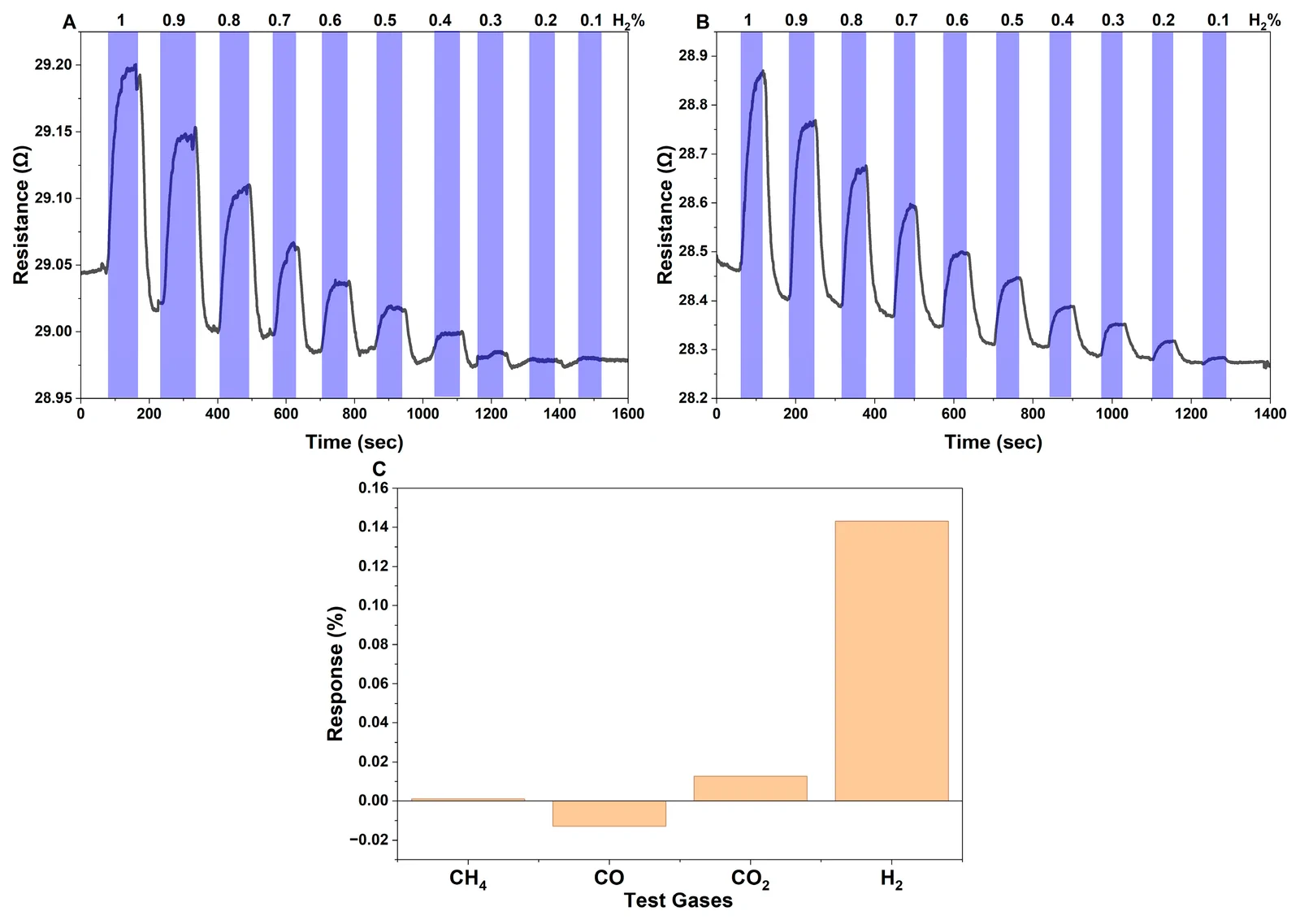
Hydrogen-sensing performance of the Pd_5_Pt_8_ NR/NS sensor under different environmental conditions. Purple shaded regions indicate approximately the time periods when H2 was flowing at the indicated concentration. Dynamic resistance responses measured with the sensor operated at 50 °C using (**A**) gas humidified to 80% relative humidity at 25 °C and (**B**) dry gas, for H_2_ concentrations decreasing from 1.0 to 0.1 vol%. (**C**) Selectivity of the Pd_5_Pt_8_ NR/NS sensor toward H_2_ compared with CH_4_, CO, and CO_2_, with each gas tested separately at 1000 ppm.

Pd-based H_2_ sensors are generally sensitive to humidity, and mild heating or drying of the sampled gas may be required to mitigate this effect. All sensing measurements presented in Figure 4 were performed under dry conditions. Preliminary measurements showed that the H_2_ response at 25 °C was strongly suppressed when the incoming gas was humidified to 80% relative humidity at 25 °C. Figure 5 therefore presents representative measurements obtained with the Pd_5_Pt_8_ NR/NS sensor operated at 50 °C under both humidified and dry gas conditions. Mild heating to 50 °C restored a measurable H_2_ response under humidified conditions. This improvement is attributed primarily to the elevated operating temperature rather than to an intrinsic humidity-independent behavior of the Pd_5_Pt_8_ composition. Increased temperature is expected to reduce competitive adsorption of water and promote water desorption from the PdPt surface, thereby maintaining surface sites available for H_2_ adsorption and dissociation. Because a systematic comparison of humidity effects across the different PdPt compositions was not performed, these results should be considered a representative demonstration that mild heating can mitigate humidity-induced suppression of the H_2_ response.

The selectivity of the Pd_5_Pt_8_ NR/NS sensor was also evaluated against common interfering gases. As shown in Figure 5C, the sensor exhibited a substantially larger response to H_2_ than to CH_4_, CO, or CO_2_ when each gas was tested separately at 1000 ppm. The responses to CH_4_ and CO_2_ were negligible, while CO produced only a small response relative to H_2_. These results demonstrate preferential sensitivity of the Pd_5_Pt_8_ NR/NS sensor toward H_2_ relative to the investigated interfering gases.

**Figure 6:**
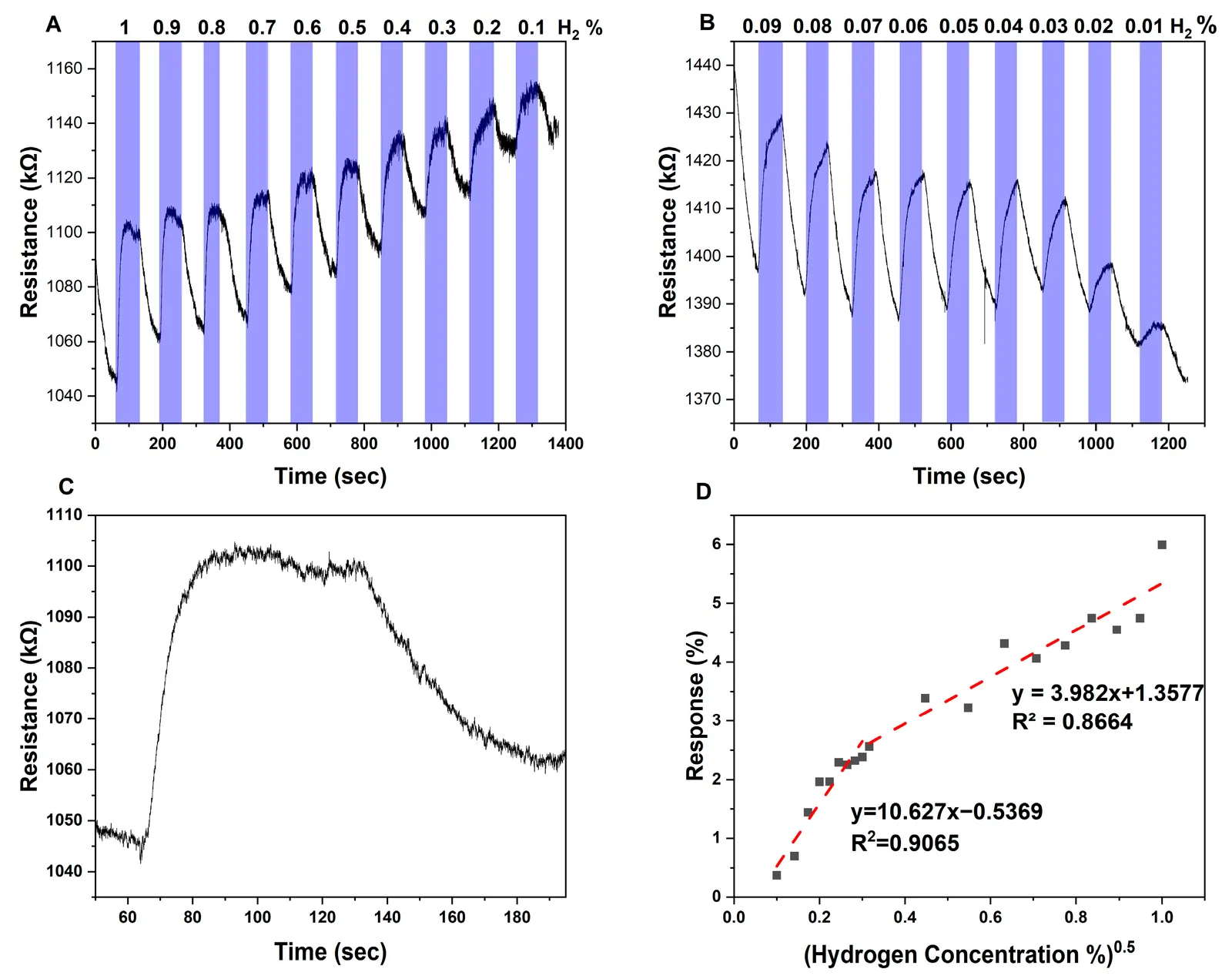
H_2_-sensing performance of a representative high-resistance Pd_5_Pt_8_ NR/NS IDE-based sensor at 25 °C. Purple shaded regions indicate approximately the time periods when H_2_ was flowing at the indicated concentration. Dynamic response to (**A**) 1.0–0.1 vol% H_2_ in air and (**B**) 0.1–0.01 vol% H_2_ in air. (**C**) Representative response to 1 vol% H_2_, from which a response time of 16 s was obtained. (**D**) Sensor response as a function of the square root of H_2_ concentration; dashed lines represent linear fits over the indicated concentration ranges.

Although the sensors shown in Figure 4 exhibited promising H_2_-sensing performance, the representative Pd_5_Pt_8_ device had a relatively low baseline resistance of approximately 14 Ω. Increasing the device resistance allows operation at lower current while maintaining a measurable voltage signal, which can reduce power consumption. We therefore fabricated Pd_5_Pt_8_ devices with lower NR/NS loading, increasing the baseline resistance to approximately 1400 Ω. Figure 6 shows the H_2_-sensing performance of a representative high-resistance Pd_5_Pt_8_ device over concentration ranges of 1.0–0.1 vol% (Figure 6A) and 0.1–0.01 vol% (Figure 6B) in air at 25 °C.

The variation in baseline resistance between successive H_2_ exposures is attributed primarily to incomplete recovery during the 1 min dry-air purge interval. Although the estimated gas residence time in the chamber is less than 6 s, indicating rapid replacement of H_2_-containing gas by dry air, hydrogen removal from the PdPt NR/NS sensing material occurs more slowly. After H_2_ is removed from the chamber, absorbed hydrogen must diffuse out of the sensing material and desorb from the surface before the sensor fully returns to its initial state. Consequently, the 1 min purge interval was insufficient for complete baseline recovery between successive exposures, resulting in the gradual baseline drift observed in Figure 6A. Importantly, this short-term incomplete recovery does not indicate irreversible sensing behavior. The closely overlapping responses obtained during decreasing and increasing H_2_ concentrations in the hysteresis experiment (Figure S8) support the reversibility of the H_2_-sensing response. The sensor exhibited a measurable response down to 100 ppm H_2_, the lowest concentration tested in this study. Figure 6C shows the response to 1 vol% H_2_, from which a response time of 16 s was obtained, consistent with that observed for the higher-loading Pd_5_Pt_8_ device.

The high-resistance device exhibited a larger response than the low-resistance device while maintaining a positive resistance response over the full concentration range tested. This indicates that, despite the lower NR/NS coverage and reduced number of conductive pathways, the device response remained dominated by changes in the intrinsic resistance of the PdPt NR/NSs rather than by changes in interparticle contact resistance. In Figure 6D, the sensor response is plotted as a function of the square root of H_2_ concentration and fitted linearly over two concentration ranges. For dissociative H_2_ uptake, the equilibrium hydrogen concentration in a metal hydride is approximately proportional to the square root of the gas-phase H_2_ concentration, consistent with Sieverts’ law. Because the sensor resistance is not necessarily directly proportional to the hydrogen concentration within the alloy over a broad concentration range, a single linear relationship is not expected across the entire range. Nevertheless, the piecewise linear fits provide a useful empirical calibration of sensor response versus H_2_ concentration.

### 3.3. Sensing Mechanism 

**Figure 7:**
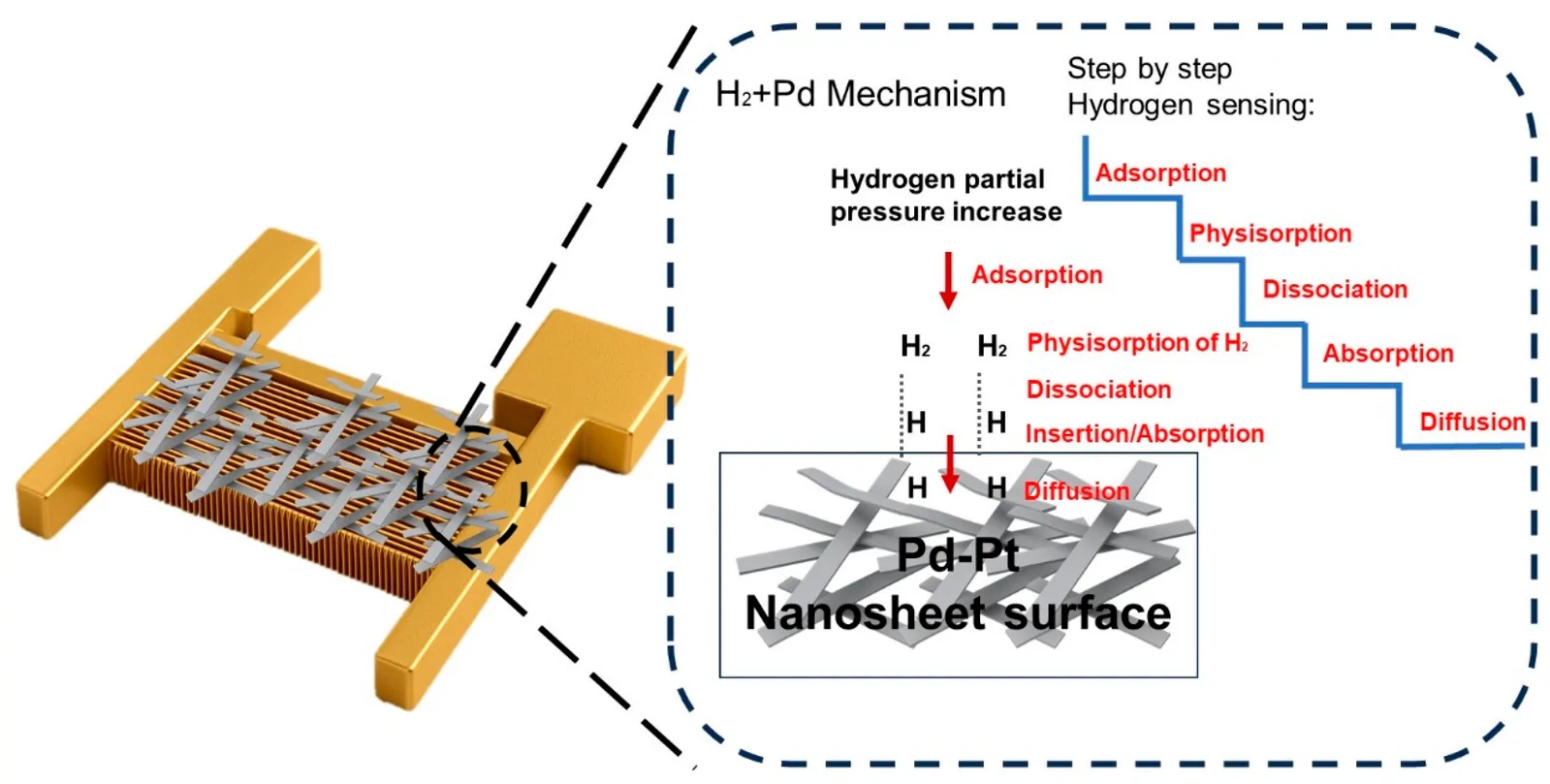
Schematic illustration of the H_2_ sensing mechanism for a PdPt alloy NR/NS IDE-based sensor.

H_2_ detection in Pd-based chemiresistive sensors originates from hydrogen adsorption, dissociation, absorption, and diffusion into the Pd-containing lattice, as illustrated schematically in Figure 7. Hydrogen incorporation modifies the electronic structure and lattice dimensions of the Pd-based material, producing a measurable change in electrical resistance [1,42]. In nanostructured networks, hydride-induced lattice expansion can also modify the contact area between neighboring structures. In some Pd nanowire networks, this contact effect decreases the overall device resistance and can produce a negative or non-monotonic response. In the present PdPt NR/NS sensors, however, the resistance increased monotonically with H_2_ concentration. The relatively large inter-sheet contact areas of the NR/NS network likely reduce the influence of contact-resistance changes, allowing the intrinsic resistance increase associated with hydrogen uptake to dominate.

The H_2_-sensing process can be represented by a sequence of adsorption, dissociation, absorption, and bulk diffusion steps. Molecular hydrogen is first adsorbed on the PdPt NR/NS surface:

**H_2_(g)** **⇌ H_2_(ads)**

Adsorbed H_2_ subsequently dissociates on available Pd and Pt surface sites:

**H_2_(ads) → 2H(ads)**

The resulting atomic hydrogen is then absorbed into the Pd-rich alloy lattice:

**H(ads)** **⇌ H(abs)**

followed by diffusion into the bulk PdPt NR/NS and formation of a hydrogen-containing solid solution or hydride phase:

**Pd_1−_ᵧPtᵧ + *x*H(abs)** **⇌ Pd_1−_ᵧPtᵧH_x_**

At relatively low hydrogen activity, hydrogen is primarily accommodated within an α-phase solid solution, whereas increasing hydrogen activity promotes formation of a more hydrogen-rich hydride phase. Hydrogen incorporation increases electron scattering and is accompanied by lattice expansion, both of which can influence electrical transport through the PdPt NR/NS network. In the present sensors, these effects produce the experimentally observed increase in resistance upon H_2_ exposure.

Pt incorporation is expected to influence the kinetics of hydrogen uptake by providing active sites for H_2_ dissociation. Atomic hydrogen generated at Pt- and Pd-containing surface sites can subsequently migrate into the Pd-rich alloy lattice. This catalytic contribution is consistent with the faster response observed for several PdPt NR/NS compositions relative to pure Pd. At the same time, Pd provides the principal hydrogen-absorbing component of the alloy. The sensing behavior therefore depends on Pd/Pt composition, reflecting a balance between H_2_ dissociation kinetics and hydrogen uptake within the Pd-rich lattice. This interpretation is consistent with the favorable performance of Pd_5_Pt_8_ and Pd_7_Pt_6_, whereas the highly Pt-rich Pd_3_Pt_10_ composition exhibited little measurable H_2_ response. This interpretation is also consistent with previous reports showing that Pt can accelerate H_2_ dissociation on Pd-based nanostructures [17,26].

During recovery, removal of H_2_ from the gas phase shifts these processes in the reverse direction. Hydrogen diffuses from the bulk toward the surface, the hydride decomposes, and surface hydrogen atoms recombine and desorb as molecular H_2_:

**2H(ads) → H_2_(g)**

The resistance therefore moves back toward its initial value. The comparatively slower hydrogen desorption and redistribution processes also help explain the incomplete baseline recovery observed during short air-purging intervals.

Table 1 compares the room-temperature H_2_-sensing performance of the present PdPt NR/NS sensors with representative previously reported Pd- and PdPt-based sensors. Because sensor response depends strongly on material morphology, device architecture, operating conditions, and response definitions, the comparison is intended to provide context rather than establish performance superiority. The present PdPt NR/NS sensors combine relatively rapid response and recovery with a monotonic positive resistance response at room temperature. In particular, the representative Pd_5_Pt_8_ sensor exhibited a response time of approximately 16 s to 1 vol% H_2_ and a measurable response down to 100 ppm H_2_, while requiring no UV-ozone post-treatment. To the best of our knowledge, this work represents the first application of this type of alloyed PdPt nanoribbon/nanosheet architecture for chemiresistive H_2_ sensing. In contrast to our previously reported Pt-decorated Pd nanowires, Pd and Pt are incorporated within the alloy NR/NS structure in the present system. The combination of Pd–Pt alloying and the two-dimensional NR/NS morphology therefore provides a simple route to room-temperature H_2_ sensing with a monotonic positive response.

The Pd_5_Pt_8_ NR/NS sensor also retained H_2_-sensing functionality during measurements performed up to 21 days after fabrication (Figure S9), indicating good storage stability under normal laboratory conditions. These measurements do not, however, constitute dedicated mechanical durability testing; vibration, mechanical shock, and quantitative adhesion measurements would be required to establish mechanical robustness for practical deployment. Device-to-device reproducibility was further evaluated using an independently fabricated Pd_5_Pt_8_ NR/NS sensor (Figure S10). The independently prepared device exhibited consistent concentration-dependent H_2_ responses over the 10,000–100 ppm range, supporting the reproducibility of the fabrication procedure and overall sensing behavior.

The values reported for this work in Table 1 were obtained from representative sensors and may differ slightly from those of the specific devices shown in Figure 4 because of device-to-device variation among independently fabricated electrodes.

## 4. Conclusions

Pd and PdPt nanoribbon/nanosheet (NR/NS) chemiresistive H_2_ sensors were fabricated by wet-chemical coreduction of Pd and Pt precursors followed by simple drop-casting onto interdigitated electrodes. The PdPt NR/NS sensors exhibited monotonic positive resistance responses to H_2_ without requiring UV-ozone post-treatment or incorporation of conductive additives. Among the investigated compositions, sensors prepared from Pd_7_Pt_6_ and Pd_5_Pt_8_, corresponding to precursor Pd:Pt mass ratios of 7:6 and 5:8, respectively, provided a favorable combination of response magnitude and response time.

The optimized Pd_5_Pt_8_ composition exhibited a response time of approximately 16 s to 1 vol% H_2_ at room temperature and produced measurable responses down to 100 ppm H_2_, the lowest concentration tested. Reducing the NR/NS loading increased the device resistance while preserving a positive H_2_ response over the investigated concentration range. The sensor also exhibited a preferential response toward H_2_ relative to CH_4_, CO, and CO_2_, while mild heating to 50 °C mitigated the strong suppression of response observed under humid conditions. Measurements performed with an independently fabricated Pd_5_Pt_8_ sensor supported device-to-device reproducibility, and sensing functionality was retained during measurements performed up to 21 days after fabrication.

The observed sensing behavior is consistent with hydrogen dissociation on Pd/Pt surface sites, absorption into the Pd-rich alloy, and formation of a hydrogen-containing phase with increased electrical resistance. Pt incorporation promotes H_2_ dissociation, while the two-dimensional NR/NS morphology provides relatively large inter-sheet contact areas that help maintain a monotonic positive resistance response in the drop-cast sensing network. These results demonstrate the potential of PdPt NR/NS architectures for low-power H_2_ sensing at or near room temperature.

## Figures and Tables

**Table 1:** Response and recovery time comparison of this work and recently published research.

Material Used in Research	Recovery Time (s)	Response Time (s)	Hydrogen Concentration %	Response %	Reference
Pd-decorated crumpled reduced graphene oxide ball (Pd-CGB)	36.5	63	1	14.8	[42]
Pd NS	50	60	1	0.4	[11]
PdMOY on IDE	60	72	1	0.5	[11]
PdMOY on paper	73	40	1	19	[11]
Pd aerogel	60	36	1	12	[12]
Pt-Pd-rGO	72	180	1	36	[41]
Nanoporous Pd-rGO	120	360	1	30	[41]
Pd NP-rGO	360	30	1		[43]
Pd NP-G	490	40	1	30	[44]
Pd_5_Pt_8_ NR/NS	22	16	1	0.12	this work
Pd_7_Pt_6_ NR/NS	26	17	1	0.42	this work
Pd_9_Pt_4_ NR/NS	10	24	1	0.04	this work
Pd_11_Pt_2_ NR/NS	5.44	25	1	0.07	this work
Pd_13_Pt_0_ NR/NS	32.5	26	1	0.24	this work
Polycrystalline PdNW	4.9	10.6	1	4.59	[10]
Quasi single crystalline PdNW	9.3	13	1	8.17	[10]
Pt thin film coated PdNW	~30	~60s	1	~4–5	[26]
Pt/Pd core–shell–graphene hybrid film	72	180	1	36	[41]
Pd@Pt@yarn thin films coated via DC sputtering	124 20	12 44	4 2	NA NA	[28]

## Data Availability

The original contributions presented in this study are included in the article/Supplementary Material. Further inquiries can be directed to the corresponding author.

## Supplementary Materials

The following supporting information can be downloaded at: https://www.mdpi.com/article/10.3390/s26196231/s1, Figures S1–S6, EDS elemental mapping and quantitative composition analyses of the Pd and PdPt NR/NS samples; Figure S7, thermogravimetric analysis of the Pd_5_Pt_8_ NR/NS sample; Figure S8, hysteresis evaluation of a representative Pd_5_Pt_8_ NR/NS sensor; Figure S9, 21-day storage-stability measurements of a Pd_5_Pt_8_ NR/NS sensor; and Figure S10, device-to-device reproducibility measurements using an independently fabricated Pd_5_Pt_8_ NR/NS sensor.

## Abbreviations

The following abbreviations are used in this manuscript:

acac	Acetylacetonate
EDS	Energy-dispersive X-ray spectroscopy
NR	Nanoribbon
NS	Nanosheet
NW	Nanowire
SEM	Scanning electron microscopy
TEM	Transmission electron microscopy
