# Supplementary material for: Palladium–Platinum Alloy Nanoribbon/Nanosheets for Room-Temperature Hydrogen Detection
Source: Sensors (Basel). 2026 Sep 30;26(19):6231. doi: 10.3390/s26196231 (PMC13667716; doi:10.3390/s26196231)
Supplement: Supplementary file 1 [file sensors-26-06231-s001.zip › sensors-4527872-supplementary.pdf]

Supporting Information for:

Palladium-Platinum Alloy Nanoribbon/Nanosheets for Room-Temperature Hydrogen Detection

Sadaf Mohsenifard [1], Thomas Thundat [1,2] and Mark T. Swihart [1,2,*]

[1] Department of Chemical and Biological Engineering, University at Buffalo (SUNY), Buffalo New York 14260, USA
[2] RENEW Institute, University at Buffalo (SUNY), Buffalo New York 14260, USA
* Correspondence: swihart@buffalo.edu

EDS elemental mapping and quantitative composition analyses for all samples are provided in Figures S1–S6.

Sample $Pd_{13}Pt_0$:

A

Element	Weight%	Atomic%
C K	8.68	21.05
O K	0.96	1.75
Si K	68.79	71.30
Pd L	21.57	5.90
Totals	100.00	
Pt:Pd atomic ratio = 0.00		

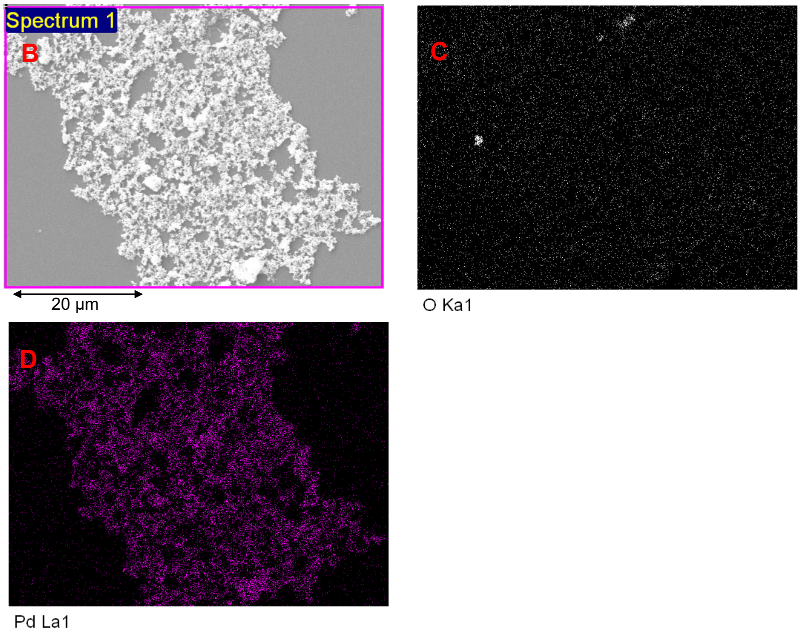

Figure S1. (A) Elemental composition table, (B) SEM image, and elemental maps of (C) oxygen and (D) palladium for the $Pd_{13}Pt_0$ NR/NS sample.

Sample $Pd_{11}Pt_2$:

A

Element	Weight%	Atomic%
C K	11.57	29.20
O K	0.95	1.81
Si K	56.20	60.64
Pd L	26.99	7.69
Pt M	4.28	0.67
Totals	100.00	
Pt:Pd atomic ratio = 0.09		

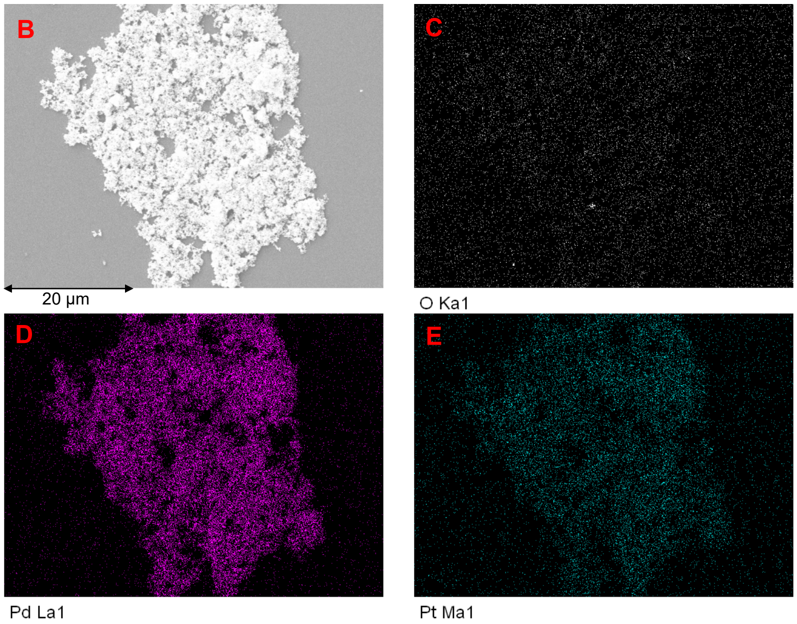

Figure S2. (A) Elemental composition table, (B) SEM image, and elemental maps of (C) oxygen, (D) palladium, and (E) platinum for the $Pd_{11}Pt_2$ NR/NS sample.

Sample Pd_9Pt_4:

A

Element	Weight%	Atomic%
C K	9.00	20.15
O K	1.19	2.00
Si K	78.90	75.51
Pd L	7.31	1.85
Pt M	3.60	0.50
Totals	100.00	
Pt:Pd atomic ratio = 0.27		

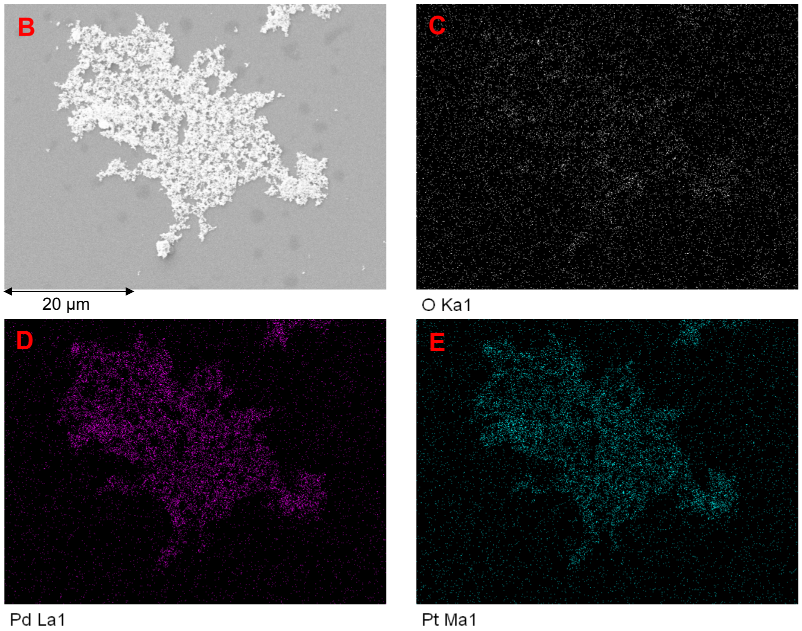

Figure S3. (A) Elemental composition table, (B) SEM image, and elemental maps of (C) oxygen, (D) palladium, and (E) platinum for the Pd_9Pt_4 NR/NS sample.

Sample Pd_7Pt_6:

A

Element	Weight %	Atomic %
C K	22.28	42.10
O K	0.47	0.67
Si K	69.29	56.00
Pd L	3.12	0.66
Pt M	4.85	0.56
Totals	100.00	
Pt:Pd atomic ratio = 0.85		

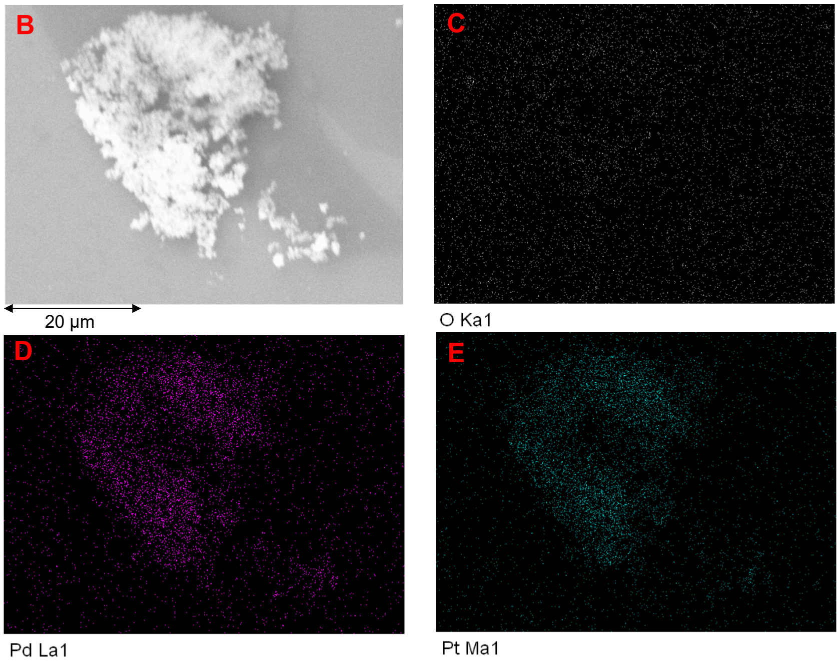

Figure S4. (A) Elemental composition table, (B) SEM image, and elemental maps of (C) oxygen, (D) palladium, and (E) platinum for the Pd_7Pt_6 NR/NS sample.

Sample Pd_5Pt_8:

A

Element	Weight %	Atomic %
C K	16.69	33.87
O K	1.04	1.58
Si K	72.48	62.91
Pd L	4.04	0.93
Pt M	5.75	0.72
Totals	100.00	
Pt:Pd atomic ratio = 0.77		

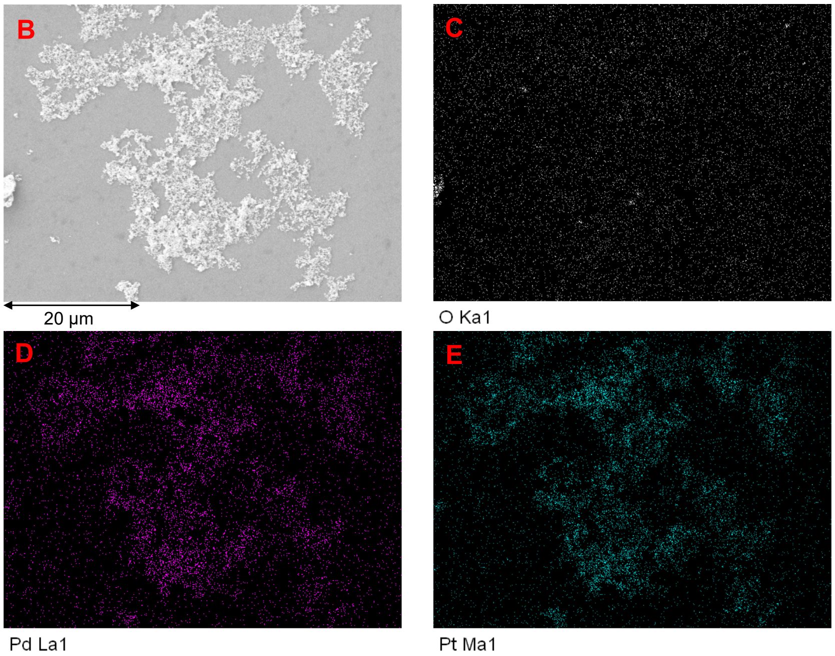

Figure S5. (A) Elemental composition table, (B) SEM image, and elemental maps of (C) oxygen, (D) palladium, and (E) platinum for the Pd_5Pt_8 NR/NS sample.

Sample Pd_3Pt_{10}:

A

Element	Weight %	Atomic %
C K	17.27	34.10
O K	1.13	1.68
Si K	74.71	63.09
Pd L	2.85	0.63
Pt M	4.04	0.49
Totals	100.00	
Pt:Pd atomic ratio = 0.78		

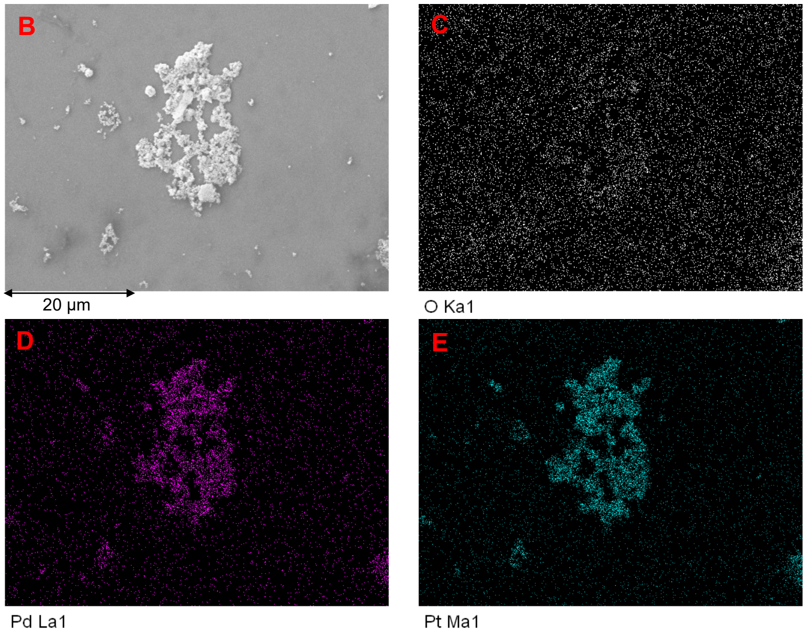

Figure S6. (A) Elemental composition table, (B) SEM image, and elemental maps of (C) oxygen, (D) palladium, and (E) platinum for the Pd_3Pt_{10} NR/NS sample.

Figure S7 presents representative thermogravimetric analysis (TGA) results for the Pd_5Pt_8 NR/NS sample under nitrogen. The sample exhibited an overall mass loss of approximately 14%. The initial mass loss below approximately 200 °C is attributed primarily to removal of adsorbed or residual solvent species, while the additional mass loss at higher temperature is consistent with decomposition of residual surface-bound organic species remaining from the synthesis.

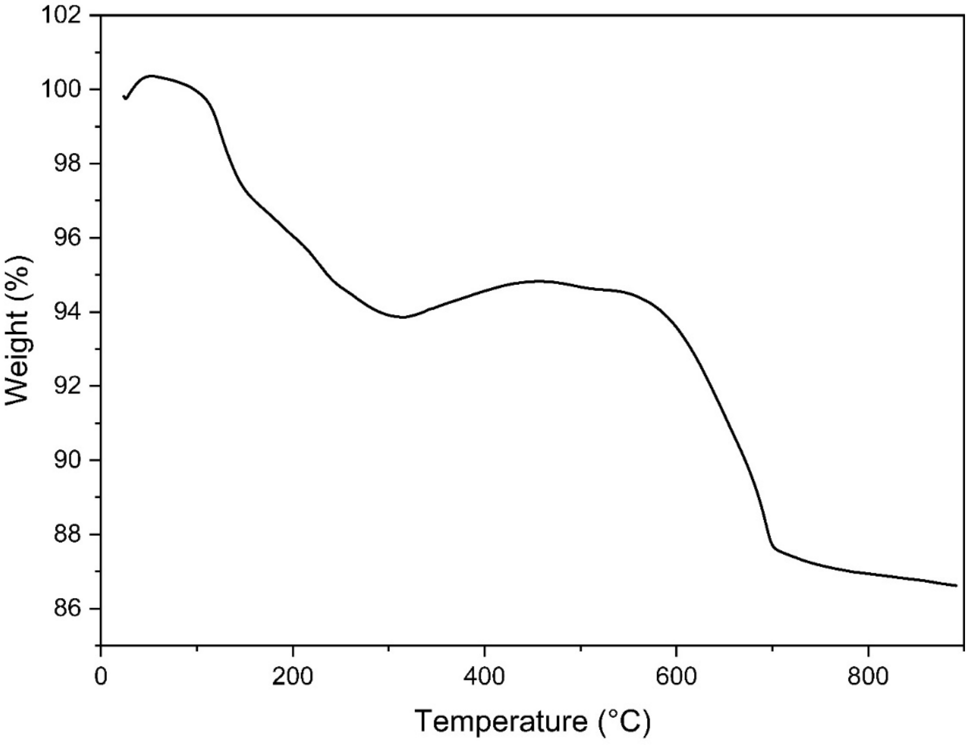

Figure S7. Thermogravimetric analysis of the Pd_5Pt_8 NR/NS sample under N_2.

Figure S8 shows the H_2 response of a representative low-resistance Pd_5Pt_8 NR/NS sensor during sequentially decreasing and increasing H_2 concentrations. The response was first measured as the H_2 concentration decreased from 10,000 ppm (1.0 vol%) to 1,000 ppm (0.1 vol%) and was then measured as the concentration increased back to 10,000 ppm. The closely overlapping responses obtained during the decreasing and increasing concentration sequences indicate minimal hysteresis over the investigated concentration range.

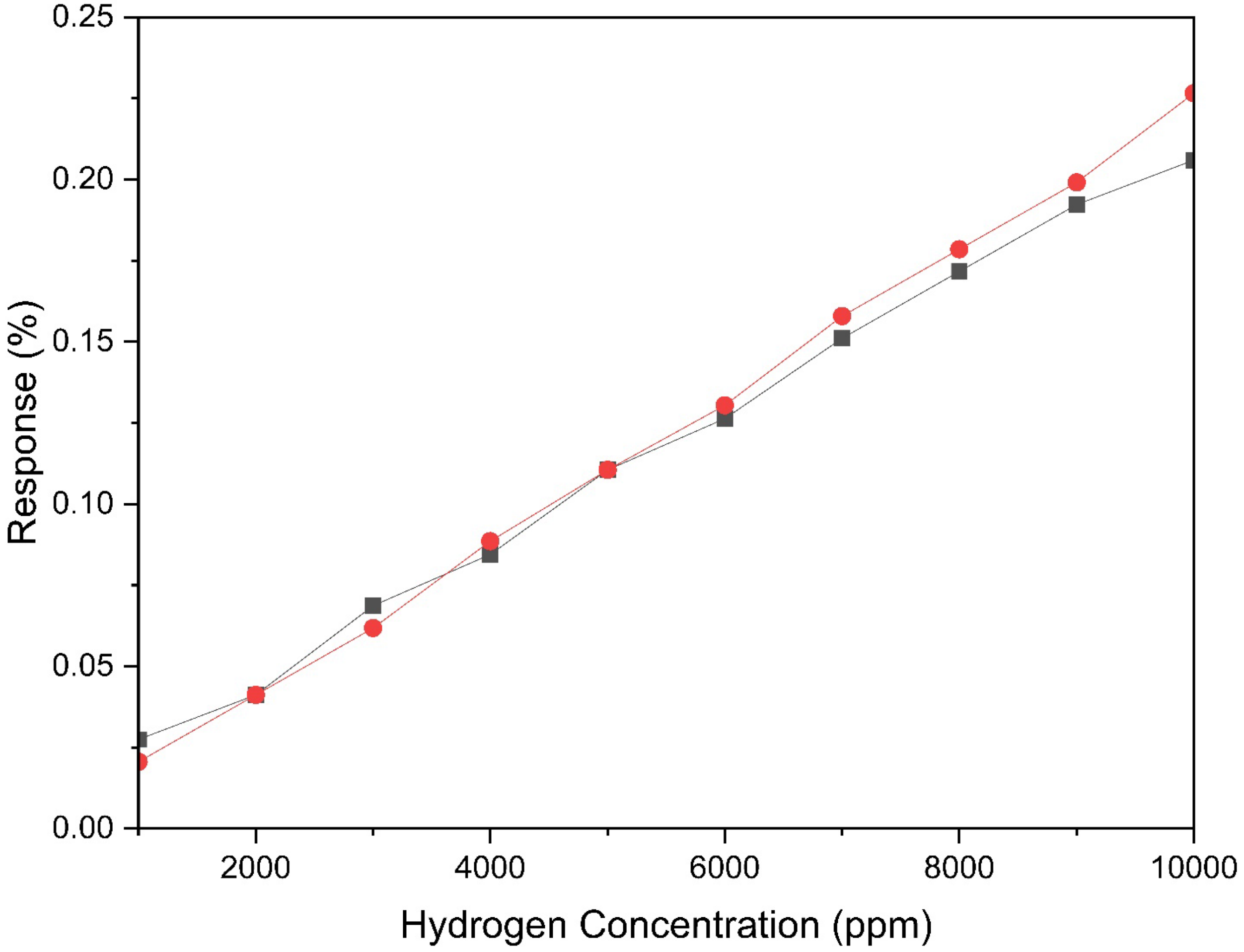

Figure S8. Hysteresis evaluation of a representative Pd_5Pt_8 NR/NS sensor at 25 °C. Sensor response during decreasing (black squares) and increasing (red circles) H_2 concentrations from 10,000 to 1,000 ppm in air. This device was different from the one shown in Figure 4 and exhibited a somewhat higher response.

Figure S9 evaluates the storage stability of a representative Pd_5Pt_8 NR/NS sensor over 21 days. H_2-sensing measurements were performed at 25 °C immediately after sensor fabrication and again after 7, 14, and 21 days of storage. The sensor retained clear concentration-dependent responses to H_2 over the 10,000–100 ppm (1.0–0.01 vol%) range at each measurement time, demonstrating retention of H_2-sensing functionality over the 21-day period.

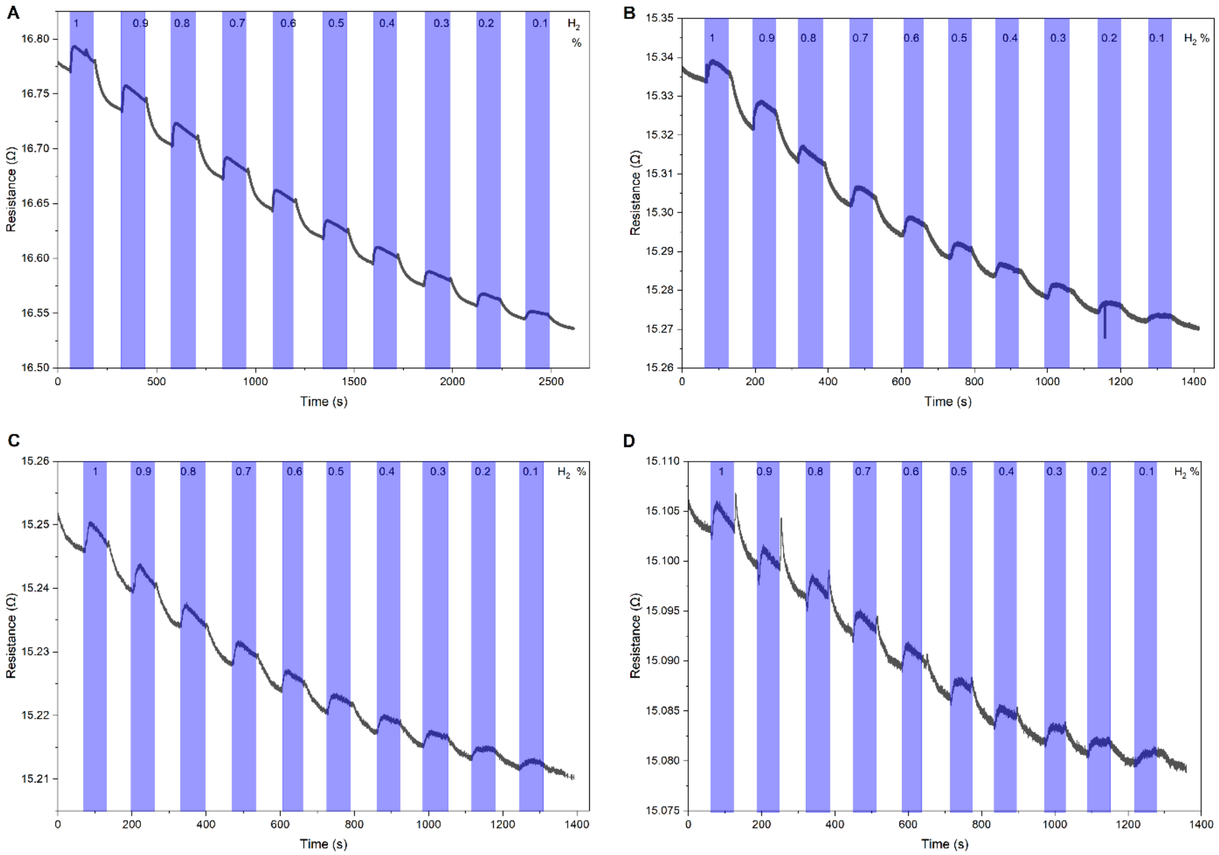

Figure S9. Storage stability of a representative Pd_5Pt_8 NR/NS sensor measured at 25 °C over H_2 concentrations from 10,000 to 100 ppm (1.0–0.01 vol%): (A) immediately after fabrication, (B) after 7 days, (C) after 14 days, and (D) after 21 days.

Figure S10 evaluates device-to-device reproducibility using an independently fabricated Pd_5Pt_8 NR/NS sensor. The sensor was tested at 25 °C over H_2 concentration ranges of 10,000–1,000 ppm (1.0–0.1 vol%) and 1,000–100 ppm (0.1–0.01 vol%). The independently prepared device exhibited clear and consistent concentration-dependent H_2 responses over both concentration ranges. Although the absolute response magnitude may vary between independently fabricated electrodes, the overall sensing behavior and concentration dependence were reproducible.

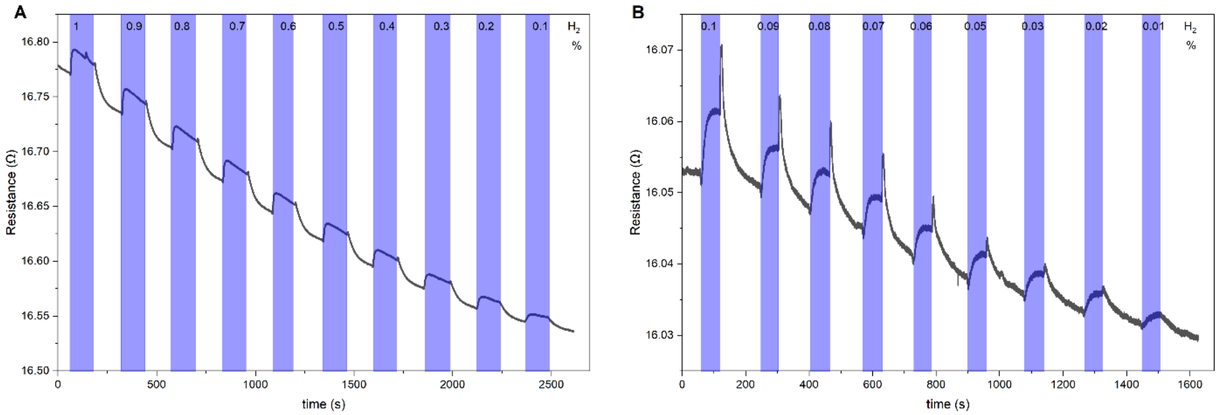

Figure S10. Device-to-device reproducibility of a Pd_5Pt_8 NR/NS H_2 sensor. Dynamic responses of an independently fabricated sensor measured at 25 °C over H_2 concentration ranges of (A) 10,000–1,000 ppm (1.0–0.1 vol%) and (B) 1,000–100 ppm (0.1–0.01 vol%).
